# Inhibition of Vps34 and p110δ PI3K Impairs Migration, Invasion and Three-Dimensional Spheroid Growth in Breast Cancer Cells

**DOI:** 10.3390/ijms23169008

**Published:** 2022-08-12

**Authors:** Marzia Di Donato, Pia Giovannelli, Antimo Migliaccio, Antonio Bilancio

**Affiliations:** Department of Medicine Precision, “Luigi Vanvitelli”, Affiliation University of Campania, Via L. De Crecchio 7, 80138 Naples, Italy

**Keywords:** breast cancer, phosphoinositide 3-kinase (PI3K), p110δ, Vps34, PI3K inhibitors, signalling, three-dimensional (3D) tumour spheroids, cell growth, cell migration, invasiveness

## Abstract

Breast cancer is a heterogeneous disease that represents the most common cancer around the world; it comprises 12% of new cases according to the World Health Organization. Despite new approaches in early diagnosis and current treatment, breast cancer is still the leading cause of death for cancer mortality. New targeted therapies against key signalling transduction molecules are required. Phosphoinositide 3-kinase (PI3K) regulates multiple biological functions such as proliferation, survival, migration, and growth. It is well established that PI3K isoform-selective inhibitors show fewer toxic side effects compared to broad spectrum inhibition of PI3K (pan-PI3K inhibitors). Therefore, we tested the PI3K p110δ-selective inhibitor, IC87114, and Vps34-selective inhibitor, Vps34-IN1, on the breast cancer cell lines MCF-7 and MDA-MB-231, representing hormone-responsive and triple-negative breast cancer cells, respectively. Our data show that both inhibitors decreased migration of MCF-7 and MDA-MB-231 cells, and Vps34 also significantly impacted MCF-7 cell proliferation. Three-dimensional (3D) in vitro culture models show that IC87114 and Vps34-IN1 treatment reduced the growth of MCF-7 and MDA-MB-231 cells in 3D tumour spheroid cultures. This study identifies IC87114 and Vps34-IN1 as potential therapeutic approaches in breast cancer.

## 1. Introduction

Breast cancer (BC) is the most common tumour in women in Western countries, despite significant improvements in diagnosis and treatment. It is noted that in countries where breast cancer incidence rates have historically been low, such as high-income Asian countries (Japan and the Republic of Korea) or emerging economies such as Africa, Asia, and South America, there has been a rapid increase in incidence rates [1]. The reason for this is unclear. The presence of the estrogen receptor (ER) on breast tumours has been shown to be a good biomarker to identify those patients who respond to anti-estrogenic therapy such as tamoxifen or lowering of circulating estrogen levels with aromatase inhibitors (e.g., anastrozole, letrozole or exemestane). Therefore, classical ER activation aimed at regulating estrogen genes may not be an exclusive mechanism. Accumulating evidence from our laboratory and others suggests that rapid, non-nuclear ER signalling pathways regulate different biological processes such as differentiation, proliferation, survival, and migration in target cells [2,3,4]. However, more than 30–50% of ER-positive (ER+) BCs are or become resistant to endocrine therapy [5,6]. Endocrine resistance occurs due to both de novo and acquired resistance. In the first case, BC develops resistance at the beginning of treatment, while acquired resistance occurs when BC is no longer responsive to endocrine therapy [6]. Mechanisms explaining the endocrine resistance range from alteration of estrogen receptors to mutations able to hyperactivate the rapid signalling controlled by ER [7,8] such as the mitogen-activated protein kinase (MAPK) [9] or the phosphoinositide 3-kinase/protein kinase B (PI3K/Akt) pathways [10,11]. Therefore, understanding the complexity of the non-genomic estrogen network in cancer is crucial to reduce BC recurrence and increase relapse-free survival. In addition, in a particular subtype of BC, the ER is not expressed. This type of BC, called triple-negative BC (TNBC), does not even express the progesterone receptor (PR) and does not exhibit human epidermal growth factor receptor 2 (HER2−) overexpression/amplification [12,13]. TNBC accounts for about 10–20% of all BCs, and only 77% of women affected by TNBC survive five years after diagnosis. Currently, chemotherapy represents the main therapeutic option in all stages of TNBC [14], and alternative treatments are not available. In this regard, ongoing clinical trials are recruiting patients for testing new drugs targeting alternative biomarkers (e.g., the effectors of Ras-dependent pathways [15], poly-ADP ribose polymerase (PARP) [16], neurotrophic receptor tyrosine kinase 1,2,3 (NTRK1/2/3), c-ros oncogene 1 (ROS1), anaplastic lymphoma kinase (ALK) [17], and the signalling effectors of the PI3K [18], used as monotherapies or combinatorial approaches for the management of BC and TNBC patients. The identification of new predictive response biomarkers and therapeutics is needed for the clinical management of both BC and TNBC patients.

The PI3K pathway is frequently mutationally activated in BC, in particular through mutations in *PIK3CA*, the gene encoding the p110α isoform of PI3K [19]. Recently, the p110α inhibitor, alpelisib, was approved for HER2−, ER+ breast cancer, in combination with the ER degrader fulvestrant. Efforts are underway to generate *PIK3CA* mutant-selective small molecule inhibitors [20,21]. In the current study, we tested the effect of interference with non-p110α PI3Ks by using two specific inhibitors, IC87114 and Vps34-IN1, on a steroid-responsive cell line (MCF-7) and in a TNBC cell line (MDA-MB-231). IC87114 is a selective inhibitor of p110δ PI3K [22,23], while Vps34-IN1 is a selective inhibitor of Vps34 class III of PI3K [24,25]. p110δ belongs to class IA PI3Ks and consists of three isoforms (p110α, p110β, p110δ) associated with a small regulatory subunit (collectively called p85), while class IB has only one isoform (p110γ) bound to the p84/p101 regulatory subunit [26]. The Vps34 (vacuolar protein sorting 34) is a sole class III PI3K member [26]. Migration is at the basis of metastases processes, the leading cause of cancer mortality. The mechanisms of cell movement are of utmost relevance for targeted intervention. Our data suggest that both p110δ and Vsp34 are involved in migration, invasiveness, and three-dimensional (3D) spheroid growth of both BC cell lines, suggesting IC87114 and Vps34-IN1 as potential powerful tools in the inhibition of BC spreading.

## 2. Results

### 2.1. Analysis of Vps34 and p110 δ Gene Expression

**Figure 1:**
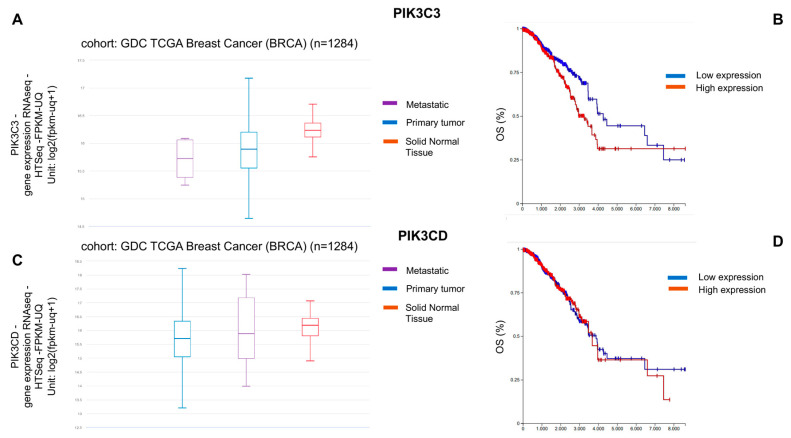
PIK3C3 and PIK3CD gene expression levels in normal mammary tissues and in BC specimens. Correlation between PIK3C3 and PIK3CD gene expression levels and overall survival (OS) in BC patients. The TCGA dataset including 1284 breast samples was used to investigate PIK3C3 and PIK3CD gene expression levels. (**A**) PIK3C3 was expressed in metastatic lesions (violet), in primary tumours (blue), and in solid normal tissue (red) at different levels (metastatic < primary tumour < solid normal tissue). (**B**) High PIK3C3 expression levels (red) were found correlated with a reduced percentage of OS in BC patients. (**C**) PIK3CD was expressed in metastatic lesions (violet), in primary tumours (blue), and in solid normal tissue (red) at different levels (primary tumour < metastatic < solid normal tissue). (**D**) High PIK3C3 expression levels (red) were found correlated with a reduced percentage of OS in BC patients.

To investigate the correlation between Vps34 (PIK3C3) and p110δ (PIK3CD) gene expression levels in normal mammary solid tissues and BC progression, data for BRCA were extracted from the Genomic Data Commons (GDC) Cancer Genome Atlas (TGCA) (Figure 1). Both genes, whose expression were analysed by RNAseq, were found highly expressed in solid normal tissues rather than in primary tumours or metastatic lesions. While PIK3C3 gene expression levels decreased with the progression of the disease passing from the primary tumours to metastatic forms (Figure 1A), PIK3CD showed the opposite trend, with higher gene expression levels in metastatic BC forms than in primary tumours (Figure 1C). However, increased expression of both PIK3C3 (Figure 1B) and PIK3CD (Figure 1D) in patients affected by BC were found highly correlated with a reduced overall survival (OS).

### 2.2. Effect of IC87114 and Vps34-IN1 on MCF-7 Cell Migration and Proliferation

**Figure 2:**
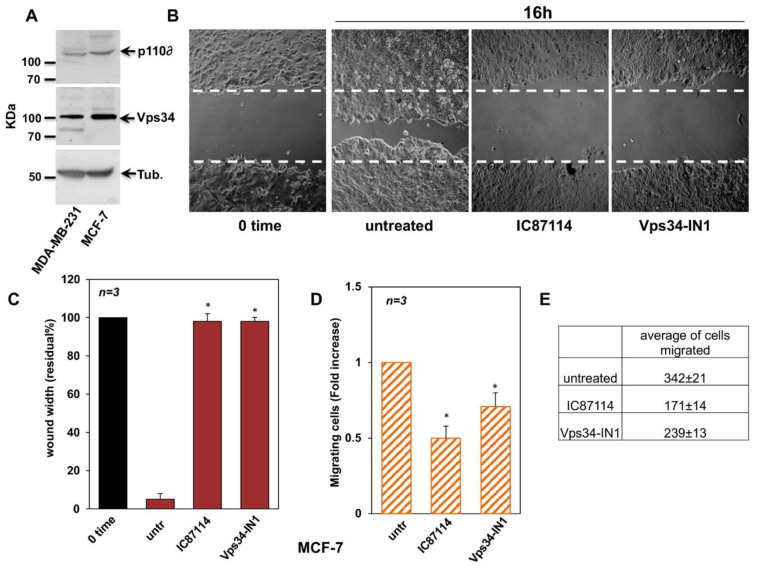
Effects of IC87114 and Vps34-IN1 on MCF-7 cell migration. (**A**) Expression of p110δ and VPs34 in total cell lysates, as assessed by immunoblotting (50 mg/lane). (**B**) Cell monolayers were wounded, and untreated or treated for the indicated times with the PI3K p110δ-selective inhibitor, IC87114, and Vps34-selective inhibitor, Vps34-IN1. Phase-contrast images are representative of three different experiments. (**C**) The wound area was measured using Image J Software, and data are presented as residual % in wound width over the control (0 time). (**D**) A migration assay was performed using Boyden’s chambers as reported in the methods section and expressed as fold change over the basal level (untreated cells). (**E**) The average number of MCF-7 cells that migrated after 6 h in the absence or presence of the inhibitors is shown. The starting number of MCF-7 cells was 30,000 cells for all experimental points, as reported and detailed in the materials and methods section. (**B**–**D**) Data from three different experiments were collected and analysed. Means and SEMs are shown. *n* represents the number of experiments. * *p* < 0.05 for the indicated experimental points versus the corresponding untreated control.

Western blotting confirmed that MCF-7 and MDA-MB-231 cells express both p110δ and Vps34 (Figure 2A). Thus, we aimed to investigate whether p110δ PI3K and Vps34 regulate BC cell migration and proliferation. To this end, we treated MCF-7, a well-differentiated and non-invasive human BC cell line, with the chemical inhibitors IC87114 and Vps34-IN1, selective for p110δ and Vps34 PI3K, respectively. To determine whether inhibition of p110δ PI3K and Vps34 affects MCF-7 migration, we performed a wound healing assay (Figure 2B,C), a transwell migration assay using collagen-coated Boyden’s chambers (Figure 2D,E), and an invasiveness assay using Matrigel-coated Boyden’s chambers (data not shown). As shown in Figure 2B and quantified in Figure 2C, a significant impairment of MCF-7 cell migration was observed in the wound healing assay by both IC87114 and the Vps34-IN1 inhibitors compared to the control (untreated cells). The inhibitory effect of IC87114 and Vps34-IN1 on migration was confirmed by the transwell migration assay (Figure 2D). The inhibitors reduced MCF-7 cell migration by approximatively 50% and 45%, respectively, compared to control cells. The average numbers of migrated cells are represented in Figure 2E. MCF-7 cells were not able to cross the Matrigel coating in our conditions to determine invasiveness (data not shown). Next, we investigated whether IC87114 and Vps34-IN1 influence DNA synthesis in MCF-7 cells using a 5-bromo-2′-deoxyuridine (BrdU) assay (Supplementary Figure S1A). We found that Vps34-IN1 reduced DNA synthesis of MCF-7 by 25%, while the IC87114 had no effect. To confirm this result, we tested the inhibitors on proliferation of MCF-7 by WST-1 assay (Supplementary Figure S1B). Our data show that 24 h treatment with IC87114 did not affect MCF-7 proliferation, while the Vps34-IN1 treatment significantly reduced their proliferation. Furthermore, we can exclude the possible involvement of cell proliferation in our migration and invasiveness experiments, as they are performed in the presence of optimal concentrations (for our conditions) of an inhibitor of proliferation, the cytosine arabinoside (Ara-C; Supplementary Figure S1C). These results suggest that, in MCF-7 cells, p110δ regulates cell migration, while Vps34 controls cell migration and proliferation.

### 2.3. Effect of p110δ and Vps34 Inhibitors on MDA-MB-231 Cell Migration, Invasion, and Proliferation

**Figure 3:**
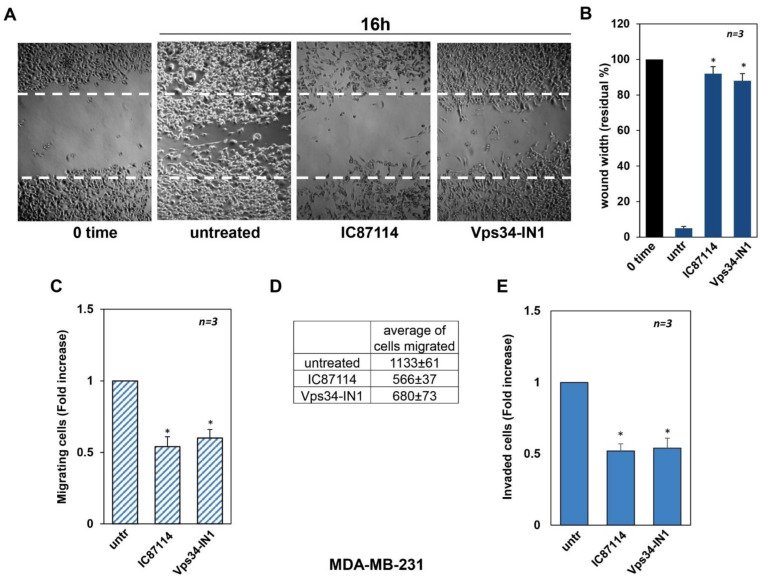
Effects of IC87114 and Vps34-IN1 on MDA-MB-231 cell migration and invasion. (**A**) cell monolayers were wounded, and untreated or treated for the 16 h with the PI3K p110δ-selective inhibitor, IC87114, and Vps34-selective inhibitor, Vps34-IN1. Phase-contrast images are representative of three different experiments. (**B**) The wound area was measured using Image J Software, and data are presented as residual % in wound width over the control (0 time). (**C**) Migration assay was performed using collagen-coated Boyden’s chambers as reported in the methods section and expressed as fold decrease over the basal level (untreated cells). (**D**) The average number of MDA-MB-231 cells that migrated after 6 h in the absence or presence of the inhibitors is shown. The starting number of MDA-MB-231 cells was 30,000 cells for all experimental points, as reported and detailed in the materials and methods section. (**E**) Invasiveness was analysed by using Matrigel-coated Boyden’s chambers. (**B**,**C**,**E**) data from three different experiments were collected and analysed. Means and SEMs are shown. *n* represents the number of experiments. * *p* < 0.05 for the indicated experimental points versus the corresponding untreated control.

Having established a role for p110δ and Vps34 in controlling migration and proliferation in MCF-7 cells, we next examined the effects of IC87114 and Vps34-IN1 in the TNBC MDA-MB-231 cell line. For this purpose, we performed a wound healing assay in the absence or presence of IC87114 and Vps34-IN1 selective inhibitors. Ten hours after the wound scratch, untreated MDA-MB-231 cells abundantly filled the wound area. As shown in Figure 3A and graphically represented in Figure 3B, both inhibitors significantly impaired cell migration after 16 h of treatment compared to untreated cells. To confirm these results, we performed the quantitative transwell assays using collagen- and Matrigel-coated Boyden’s chambers for analysing the migration (Figure 3C,D) and the invasiveness in MDA-MB-231 cells (Figure 3E), respectively, and observed that both inhibitors significantly reduced the cell migration and invasiveness. The average number of migrated cells is represented in Figure 3D. We then tested the effects of the inhibitors on MDA-MB-231 cell proliferation and observed no significant effects on BrdU incorporation or cell proliferation analysed by WST-1 assay (Supplementary Figure S1D,E, respectively). It is worth noting that we can exclude the possible involvement of cell proliferation in our migration and invasiveness experiments, as they are performed in the presence of optimal concentrations of an inhibitor of proliferation, cytosine arabinoside (Ara-C; Supplementary Figure S1F). Overall, these data suggest that p110δ and Vps34 regulate cell migration and invasion in TNBC MDA-MB-231 cells.

### 2.4. IC87114 and Vsp34-IN1 Impair the Growth of MCF-7- and MDA-MB-231-Derived Spheroids

**Figure 4:**
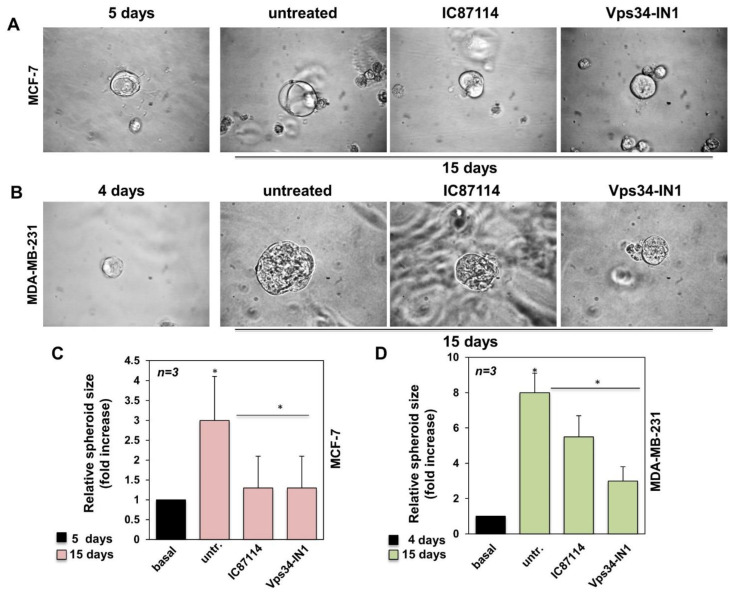
IC87114 and Vsp34-IN1 impair the 3D spheroid growth models on MCF-7- and MDA-MB-231-derived spheroids. MCF-7 (**A**) and MDA-MB-231- (**B**) derived spheroids were generated as reported in the methods section. Five (**A**) or four (**B**) days after the cells were embedded in Matrigel, representative images were acquired. Spheroids were then left untreated or treated with the PI3K p110δ-selective inhibitor, IC87114, and Vps34-selective inhibitor, Vps34-IN1, and further images were captured after 15 days. Phase-contrast images in A and B are representative of three different experiments. The MCF-7- (**C**) and MDA-MB-231- (**D**) derived spheroid size was calculated using the Leica Suite software under basal conditions (5 or 4 days, respectively) or in cells left untreated or treated for an additional 15 days with the indicated inhibitors. Final size was expressed as fold-increase in the relative spheroid size. (**C**,**D**) *n* represents the number of experiments. Means and SEM are shown. * *p* < 0.05, as compared to untreated cells.

It is well established that 3D in vitro cultures are a good model to study drug response in human cancer. Therefore, we decided to investigate the inhibitory effect of IC87114 and Vps34-IN1 on spheroids derived from MCF-7 and MDA-MB-231 cells. Cells were plated in extracellular matrix (ECM), and small, round spheroids were observed after 5 days (MCF-7, Figure 4A) or 4 days (MDA-MB-231, Figure 4B). At this point, spheroids from both cell lines were grown for an additional 15 days untreated (control) or in the presence of IC87114 or Vps34-IN1. As shown in Figure 4A and graphically represented in Figure 4C, both inhibitors significantly reduced the growth of MCF-7-derived spheroids. Both inhibitors also significantly reduced the growth of MDA-MB-231-derived spheroids, although IC87114 reduced the growth to a lesser extent than Vps34-IN1 (Figure 4B,D). Overall, these data suggest that IC87114 and Vps34-IN1 significantly inhibit 3D spheroids growth in both cell lines, although IC87144 is more effective in MCF-7. These data are supported by the finding that 3D models are preferable to 2D cultures, which cannot fully resemble the cancer dimensionality and microenvironment in the tumour mass. It is noteworthy that cancer cell migration and invasion occur through dynamic processes involving cell adhesion/migration and ECM degradation/remodelling that are strictly organized amongst themselves. Although a 3D in vitro model is not exactly comparable to the original breast tumour tissue, it provides valuable insights into the morphological and signalling behaviour of tumour cells during cell migration in a 3D microenvironment.

### 2.5. Effects of IC87114 and Vsp34-IN1 on MCF-7 Cell Signalling Pathways

**Figure 5:**
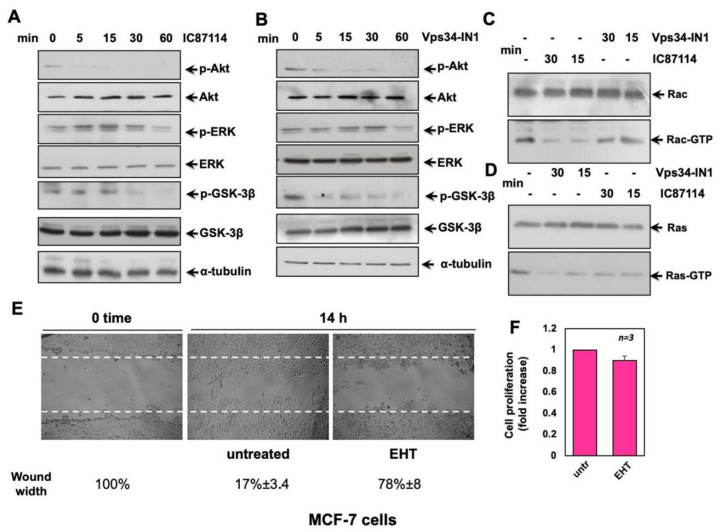
Effects of IC87114 and Vsp34-IN1 on MCF-7 cell signalling pathways. MCF-7 cells were untreated or treated for the indicated times with IC87114 (**A**) or Vsp34-IN1 (**B**), and cell lysates were analysed by western blot, using antibodies against p-Akt (p-Ser-473 Akt), p-ERK (P-Tyr 204 ERK 1 and the corresponding phosphorylated ERK 2), and p-GSK-3β (p-Ser-9 GSK-3β). The blots were re-probed using anti-ERK, anti-Akt, anti-GSK-3β, or anti-α-tubulin antibodies, as loading controls. (**C**,**D**) MCF-7 cells were untreated or treated with IC87114 and Vsp34-IN1 for 30 and 15 min, and Rac (**C**) and Ras (**D**) pull down assays were performed. Active (Rac-GTP) or total Rac (Rac) in C and active (Ras-GTP) or total Ras in D were detected by western blot. Western blots (**A**–**D**) are representative of three different experiments. (**E**) Cell monolayers were wounded, and unchallenged or challenged for 14 h with the indicated compounds. Phase-contrast images are representative of three different experiments. The wound area was measured using Image J Software, and data are presented as residual % in wound width over the control (0 time) below the corresponding images. (**F**) Cell proliferation was analysed by WST-1, and data represented as fold change over the basal level (untreated cells). Means and SEM are shown. In (**E**,**F**), EHT is EHT 1864.

Having shown that IC87114 and Vps34-IN1 inhibit BC cell migration in vitro, we next investigated the signalling effectors that could be involved in cell migration and/or proliferation by western blot analysis in a time-course assay. Cycling MCF-7 cells were untreated or treated for 5, 15, 30, or 60 min with IC87114 or Vps34-IN1, and the phosphorylation status of different molecular effectors was analysed (Figure 5). As indicated in Figure 5A, IC87114 treatment reduced the levels of p-Akt after 5 min of treatment, while extracellular signal-regulated kinase (ERK) phosphorylation was only slightly reduced after 60 min. IC87114 also reduced phosho-glycogen synthase kinase 3β (p-GSK-3β) levels after 30 min. Treatment with Vps34-IN1 had a similar effect on the examined proteins (Figure 5B). p110δ has previously been reported to positively regulate Rac1 in primary macrophages [27]. We therefore investigated whether p110δ or Vps34 play a similar role in BC cells. Pharmacological inactivation of p110δ, but not of Vps34, led to a reduction in Rac1 activation in MCF-7 (Figure 5C). Ras has been reported to regulate numerous biological effects and is mutated in many tumours. We therefore investigated the pharmacological effect of the two inhibitors on Ras-GTP loading by a Ras pull-down assay in MCF-7 cells (Figure 5D) and observed that both treatments inhibited Ras activation to the same extent. To better understand the role of Rac in MCF-7 cells, we used the specific Rac inhibitor, EHT 1864 [28], in migration and proliferation assays. Experiments presented in Figure 5E,F show that Rac is involved in MCF-7 cell migration but not proliferation, since EHT 1864 was able to inhibit the migration of cells into the wounded area (Figure 5E) but not cell proliferation analysed by WST-1 (Figure 5F). These results suggest that p110δ plays a role in migration through Akt/Rac activation, while Vps34 might exert its effects through Akt/Ras activation.

### 2.6. Effects of IC87114 and Vsp34-IN1 on MDA-MB-231 Cell Signalling Pathways

**Figure 6:**
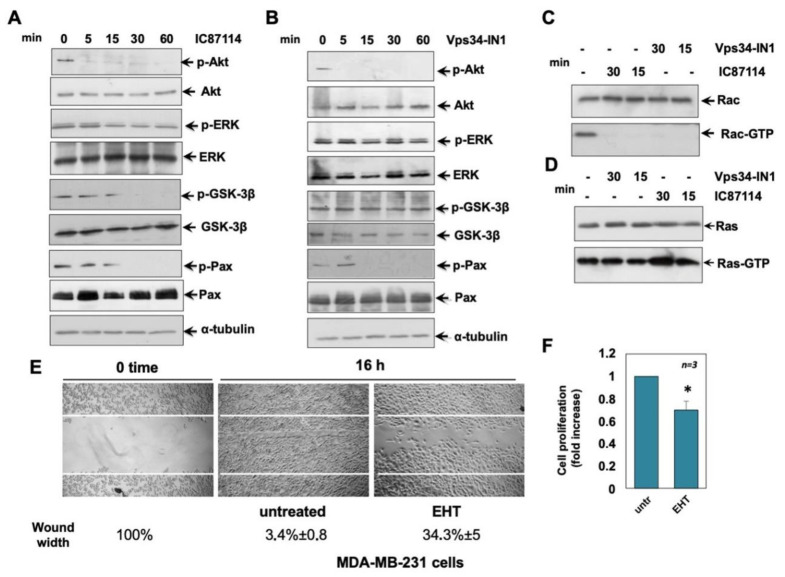
Effects of IC87114 and Vps34-IN1 on MDA-MB-231 cell signalling pathways. MDA-MB-231 cells were untreated or treated for the indicated times with IC87114 (**A**) or Vsp34-IN1 (**B**), and protein lysates were analysed by western blot, using the indicated antibodies against p-Akt (p-Ser-473 Akt), p-ERK (P-Tyr 204 ERK 1 and the corresponding phosphorylated ERK 2), p-GSK-3β (p-Ser-9 GSK-3β), and p-Pax (P-Tyr 118 Pax). The filters were re-probed using anti-ERK, anti-Akt, anti- GSK3-β, anti-Pax, or anti- α-tubulin antibodies, as loading controls. In C and D, MDA-MB-231 cells were untreated or treated with IC87114 and Vsp34-IN1 inhibitors for 30 and 15 min, and Rac (**C**) and Ras (**D**) pull down assays were performed. Active (Rac-GTP) or total Rac (Rac) in C and active (Ras-GTP) or total Ras in D were detected by western blot. Western blots (**A**–**D**) are representative of three different experiments. (**E**) Cell monolayers were wounded, and untreated or treated for 16 h with the indicated compounds. Phase-contrast images are representative of three different experiments. The wound area was measured using Image J Software, and data are presented as residual % in wound width over the control (0 time) below the corresponding photographs. (**F**) Cell proliferation was analysed by WST-1, and data represented as fold change over the basal level (untreated cells). Means and SEM are shown. (**E**,**F**) EHT stands for EHT 1864. * *p* < 0.05, as compared to untreated cells.

Based on the above observation that in MDA-MB-231 cells, p110δ PI3K and Vps34 are involved in cell migration, we next investigated possible effectors involved in these processes (Figure 6). We found that, as in MCF -7 cells, treatment of MDA-MB-231 cells with IC87114 or Vps34-IN1 led to a complete inhibition of Akt phosphorylation, while neither treatment altered ERK phosphorylation (Figure 6A,B). Treatment with IC87114 significantly reduced GSK3-β phosphorylation after 30 min (Figure 6A), while treatment with Vps34-IN1 did not significantly affect GSK3-β phosphorylation (Figure 6B). Paxillin is a scaffold/adaptor protein that, when phosphorylated at specific Tyr or Ser residues, causes the recruitment of signalling molecules involved in cell migration [29]. Paxillin is able to recruit GEFs and GAPs to focal adhesions, leading to the activation of Rho GTPases such as Cdc42 and Rac1 [29]. We therefore investigated the effect of IC87114 and Vps34-IN1 on paxillin, Rac, and Ras activation in TNBC MDA-MB-231 cells (Figure 6A–D). Treatment with IC87114 or Vps34-IN1 leads to complete inhibition of phospho-paxillin (Figure 6A,B) and Rac activation (Figure 6C), while no inhibition was observed for Ras activation (Figure 6D). To understand the role of Rac activation in MDA-MB-231 cells, we used the specific Rac inhibitor EHT 1864. Experiments presented in Figure 6E,F show that Rac is involved in MDA-MB-231 cell migration and proliferation, since EHT 1864 was able to inhibit the migration of cells into the wounded area (Figure 6E) and cell proliferation analysed by WST-1 (Figure 6F). Overall, the experiments shown in Figure 5E or 6E demonstrate that Rac is involved in migration in MCF-7 and MDA-MB-231 cell lines, albeit to a lesser extent in MDA-MB-231 cells (Figure 6E), and in proliferation only in MDA-MB-231 cells (Figure 6F). Overall, these data show that p110δ and Vsp34 play a role in TNBC MBD-MB 231 cells, independent of Ras activation.

## 3. Discussion

BC is the second leading cause of death in women, and TNBC is associated with high treatment resistance and metastatic potential. Current treatment strategies consist of endocrine therapy, targeted therapy, and chemotherapy [30]. However, de novo or acquired drug resistance is very common, leading to relapse and tumour progression. Dysregulation/hyperactivation of the PI3K/Akt/Mammalian Target of Rapamycin (mTOR) pathway, responsible for intrinsic resistance, has been found in 70% of human BCs [31]. The PI3K/Akt/mTOR signalling pathway controls many key biological functions of cells, such as proliferation, migration, and growth, which are hallmarks of cancer [26,32]. PI3K is a family of lipid kinases divided into three classes, with the IA class playing a key role in many cancers by producing the lipid messenger phosphatidylinositol-3,4,5-triphosphate (PIP3), which activates Akt by recruiting it to the plasma membrane [26]. Akt triggers the activation of mTOR, leading to protein synthesis and cell growth. The PI3K/Akt/mTOR pathway is turned off by phosphatase and tensin homologue deleted on chromosome ten (PTEN) by dephosphorylating phosphatidylinositol-3,4,5-triphosphate (PIP3) into phosphatidylinositol-4,5-triphosphate (PIP2) [26]. The mechanism of PI3K/Akt/mTOR resistance may occur at different levels in BC [30,31,33,34,35]. First, upregulation of receptor tyrosine kinase (RTK) may enhance activation of the PI3K/Akt/mTOR pathway [30]. In addition, mutations in the PIK3CA gene leading to hyperactivation of the p110α PI3K catalytic subunit have been reported to occur in 30–40% of patients with ER+ BC and in 20–25% of BC patients with overexpression of ERB2, and mutations in Akt1 and PTEN have been found in 13–24% and 7% of ER+ BC patients, respectively [31,33,35]. In addition, PI3K/Akt can phosphorylate ERa at S167 in the activation function 1 domain (AF1) in the absence of estrogen, and mitogen-activated protein kinase (MAPK) at S118 in AF1, leading to estrogen-independent ER activation [30,31,35]. Moreover, PI3K/Akt promotes chemotherapy-resistant proteins such as Multidrug Resistance Associated Protein 1 (MRP1), ABCG2, P-glycoprotein, and anti-apoptotic effects [30]. Furthermore, preclinical studies suggest that inhibition of the PI3K/Akt/mTOR pathway can restore sensitivity to endocrine therapy, anti-HER2 therapy, and chemotherapy [30,31,33,34,35]. Therefore, it is critical to find novel therapeutic strategies that can interfere with the molecular mechanisms that drive proliferation and metastasis. In this study, we report the role of p110δ PI3K and Vps34 in two different cell lines. We used MCF-7, a classical model to study hormone-dependent cell response, and the MDA-MB-231 TNBC cell line that lacks expression of estrogen receptor α (ERα), progesterone receptor (PR), and epidermal growth factor receptor 2 (HER2) and shows a high rate of metastasis [13]. We focused on the role of p110δ PI3K, and Vps34. p110δ is an isoform of the PI3K class IA, which is highly expressed in white blood cells and regulates proliferation and movement [22,36,37]. p110δ is expressed in human BC and in melanoma cells [38,39], with histological analysis of human BC patients showing a good correlation between the increase in p110δ expression and the increase in malignancy from grade I to grade III [40]. Administration of the p110δ PI3K inhibitor IC87114 in a mouse model of human BC strongly impaired cancer growth [40]. In addition, a p110δ inhibitor was also used to treat solid tumours in a mouse model [41]. These results suggest that p110δ may have direct and indirect effects on tumour growth. Several p110δ inhibitors have been tested in clinical trials [21,42,43]. Our results show that pharmacological inactivation of p110δ inhibits MCF-7 and MDA-MB-231 cell migration in scratch wound in Boyden’s chambers assay and 3D spheroid growth in 3D culture models. Vps34 belongs to the III class of PI3K, and pharmacological inhibition has been reported to mediate vesicle transport and autophagy phenomena [44,45,46]. Accumulating evidence suggests that Vps34 is involved in human cancer development [47]. In addition, the Vps34 inhibitor, Vps34-IN1, increased cytotoxic activity in MCF-7 and MDA-MB-231 cells when treated with sunitinib and erlotinib, which have shown modest activity in patients with BC [47,48]. Furthermore, targeting Vps34 converts cold to hot inflamed tumours, increasing the efficacy of blocking PD-L1/PD-1 [49]. Vps34 has been shown to play a critical role in solid and hematopoietic cancers. Thus, Vps34 induces phosphorylation of p62, which drives autophagosomes and cancer progression [50]. Inhibition of Vps34 has been shown to induce apoptosis in AML cells but not in normal CD34+ hematopoietic cells [51]. Therefore, we tested the effect of Vps34-IN1 on BC-derived MCF-7 and TNBC-derived MDA-MB-231 cells. We found that Vps34-IN1 effectively blocked migration in both cell lines and cell growth in MCF-7 cells. Mechanistically, our results suggest that p110δ and Vsp34 signalling play a key role in Rac activation. In our experimental systems, Rac is involved in migration but not proliferation of MCF-7 cells and in both processes in MDA-MB-231 cells. Rac signalling is important for lamellipodia formation and efficient cell motility. Indeed, we found decreased Rac-GTP activity upon treatment with IC87114 in MCF-7 cells and upon treatment with IC87114 or Vps34-IN1 in MDA-MB-231 cells, whereas activation of ERK and Ras-GTP levels were not affected. Moreover, these results suggest that p110δ plays a role in migration through Akt/Rac activation, whereas Vps34 may exert its effect through Akt/Ras activation. Our results suggest that p110δ PI3K is involved in the regulation of cell migration in BC steroid receptor-positive MCF-7 cells and in TNBC MDA-MB-231 cells. p110δ PI3K regulates cell migration independently of hormone status, and its inhibition (by IC87114) reduces migration in both cell lines, while leaving proliferation unaffected. In contrast, Vps34 regulates migration in both cell lines (MCF-7 and MDA-MB-231), with a clear inhibition of proliferation by Vps34-IN1 only in MCF-7 BC cells. Furthermore, IC87114 and Vps34-IN1 show growth inhibition of 3D-MCF-7 and MDA-MB-231 in spheroid models. Further studies are needed to understand the mechanism of regulation of cell migration and proliferation in MCF-7 and MDA-MB-231 cell lines. In conclusion, new drugs such as p110δ and Vps34 inhibitors may have a promising synergistic effect in addition to standard therapy to address the challenge of treatment resistance in BC.

## 4. Materials and Methods

### 4.1. Chemicals, Reagents, and Constructs

The PI3Kδ inhibitor IC87114 (Merck-Serono, Plymouth, MA, USA) was used at 5 μM (final concentration) [23]. The Vsp34 selective inhibitor (Vps34-IN1; Selleck Chemicals LLC, Houston, TX, USA) was used at 1 μM (final concentration). The Rac inhibitor (EHT 1864; Sigma Aldrich, St. Louis, MO, USA) was used at 10 μM (final concentration).

### 4.2. Cell Culture

Human BC-derived MCF-7 cells and TNBC-derived cells MDA-MB-231 were from Cell Bank Interlab Cell Line Collection (ICLC, Genova, Italy). The supplier authenticated cell lines for DNA profile using Short Tandem Repeat (STR) analysis. The cell lines employed were routinely monitored for *Mycoplasma*. Cells were maintained at 37 °C in humidified 5% CO_2_ atmosphere. Media and supplements were from Gibco (Thermo Fisher Scientific; Waltham, MA, USA). MCF-7 cells were cultured in phenol-red DMEM containing 10% foetal bovine serum (FBS), penicillin (100 U/mL), streptomycin (100 U/mL), glutamine (2 mM), hydrocortisone (3.75 ng/mL), and insulin (6 ng/mL). MDA-MB-231 cells were cultured as reported [52].

### 4.3. DNA Synthesis and WST-1 Assay

Cells on coverslips were left untreated or treated with the indicated inhibitors for 14 h. After in vitro pulse-labeling of cultured cells with BrdU (100 µM final concentration; Sigma-Aldrich, St. Louis, MO, USA), cells were fixed for 20 min with paraformaldehyde (4%, wt/vol in PBS; Merck, Saint Louis, MO, USA), permeabilized for 10 min with Tween (0.1%, vol/vol in PBS; Biorad, Hercules, CA, USA), and incubated for 1 h with PBS containing FBS (1% vol/vol). The BrdU incorporation into newly synthesized DNA was analysed using a DMLB Leica (Leica, Wetzlar, Germany) fluorescent microscope equipped with HCX PL Apo 63× oil objective, as reported [53]. Total nuclei were stained for 5 min with Hoechst 33258 (1 µg/mL; Sigma Aldrich, St. Louis, MO, USA). The BrdU incorporation was calculated by the following formula: (No. of BrdU-positive cells/No. of total cells) × 100. Cell proliferation was assayed using WST-1 reagent (Roche, Basel, Switzerland), as described [53].

### 4.4. Wound Scratch and Boyden’s Chambers Migration Assays

In wound scratch assays, 1.5 × 10^5^ cells were seeded in 24-well plates. Cells were wounded using 10 μl sterile pipette tips and left untreated or treated for the times indicated in the figures or their relative legends with the indicated inhibitors. To avoid cell proliferation, cytosine arabinoside (Ara-C, Sigma-Aldrich) at 50 μM (final concentration) was added in the cell culture medium. Different concentrations were tested (for details, see Supplementary Materials). Different fields were analysed using a DMIRB microscope (Leica) equipped with N-Plan 10× objective (Leica), as reported [38]. Phase-contrast images were captured using a DFC 450C camera (Leica) and acquired using Application Leica Suite Software. Images are representative from at least three different experiments. The wound gap was calculated using Image J Software and expressed as % of the decrease in the wound area. Migration and invasiveness assays were performed as previously reported [52,54], using collagen or Matrigel pre-coated Boyden’s chambers with 8 μm polycarbonate membrane (Corning; Corning, NY, USA) for migration or invasiveness, respectively, and 3 × 10^4^ cells for each experimental point in both assays. The indicated stimuli were added to the upper and the lower chambers. For inhibiting cell proliferation, Ara-C (at 50 μM) was included in the cell culture medium. After 6 h (for migration) or 18 h (for invasiveness), non-migrating/invading cells from the membrane upper surface were removed using a sterile cotton swab, and the membranes were fixed in paraformaldehyde, stained with Hoechst, removed with forceps, and mounted. At least 30 fields/membrane were counted using a DMBL (Leica) fluorescent microscope, equipped with HCPL Fluotar 63× objective [52,54].

### 4.5. D Culture

MCF-7 and MDA-MB-231 cells (2.5 × 10^4^) were mixed with 230 µL of phenol red-free growth factor reduced Matrigel (10 mg/mL; BD Biosciences, Erembodegem, Belgium) and 50 µL of spheroid plating medium for each well. Spheroid plating medium was made using DMEM/F12 medium, 5% FBS, 100 U/mL penicillin, 100 U/mL streptomycin, GlutaMAX 100× (Gibco), 10 mM HEPES, 1 mM nicotinamide (Merck), 1.25 mM N-acetylcysteine (Sigma-Aldrich), and 10 µM Y-27632 (Merck-Millipore, Temecula, CA, USA). Spheroids were generated as reported [55]. After 3 days, the spheroid plating medium was replaced with a similar medium in the absence of N-acetylcysteine and Y-27632. On 4th or 5th day, the spheroids were left untreated or treated for an additional 15 days with the indicated inhibitors. The medium was changed every 2 days. Different fields were analysed using a Leica DMIRB (Leica) microscope equipped with C-Plan ×40 objective (Leica), and phase-contrast images were acquired using a DFC 450C camera (Leica). The relative spheroid size was calculated using the Application Suite software (Leica) and expressed as a fold increase over the basal spheroid size, which was measured on the 4th or 5th day.

### 4.6. Protein Lysates, Rac and Ras Assays, and Western Blot

The preparation of cell lysates was performed as reported [56]. The Rac pull-down assay and the Ras pull-down assay were done using lysate proteins at 1 mg/mL and Rac and Ras activation kits (Merk-Millipore, Temecula, CA, USA), following the manufacturer’s instructions. For western blot analysis, the following antibodies were used: mouse monoclonal anti-p44 and -p42 Phospho-ERK (sc-7383; Santa Cruz, Dallas, TX, USA), anti-p42 ERK (sc-1647; Santa Cruz), anti-paxillin (clone 349; BD Biosciences), and anti-tubulin (T5168; Sigma-Aldrich) antibodies, and rabbit polyclonal anti-Phospho-Akt (Ser473) (Antibody #9271; Cell Signalling, Danvers, MA, USA), anti-Akt (#9272; Cell Signalling), anti-Phospho-Tyr 118 paxillin (BD Biosciences), anti-Phospho-GSK-3α/β (Ser21/9) (#9331; Cell Signalling) and anti-GSK-β (sc-377213; Santa Cruz) antibodies. The ECL system (GE Healthcare, Chicago, IL, USA) was used to reveal immunoreactive proteins.

### 4.7. Statistical Analysis

The presented results are expressed as the mean ± SEM of at least three independent experiments. Two-tailed unpaired Student t-tests and one-way or two-way ANOVA were used.

## Data Availability

The data presented in this study are available in this article and Supplementary Material. The datasets used and/or analyzed during the current study are available from the corresponding author on reasonable request.

## Supplementary Materials

The following supporting information can be downloaded at: https://www.mdpi.com/article/10.3390/ijms23169008/s1.
