# Supplementary material for: Inhibition of Vps34 and p110δ PI3K Impairs Migration, Invasion and Three-Dimensional Spheroid Growth in Breast Cancer Cells
Source: Int J Mol Sci. 2022 Aug 12;23(16):9008. doi: 10.3390/ijms23169008 (PMC9409264; doi:10.3390/ijms23169008)
Supplement: Supplementary file 1 [file ijms-23-09008-s001.zip › ijms-1791502-supplementary.pdf]

Supplementary material

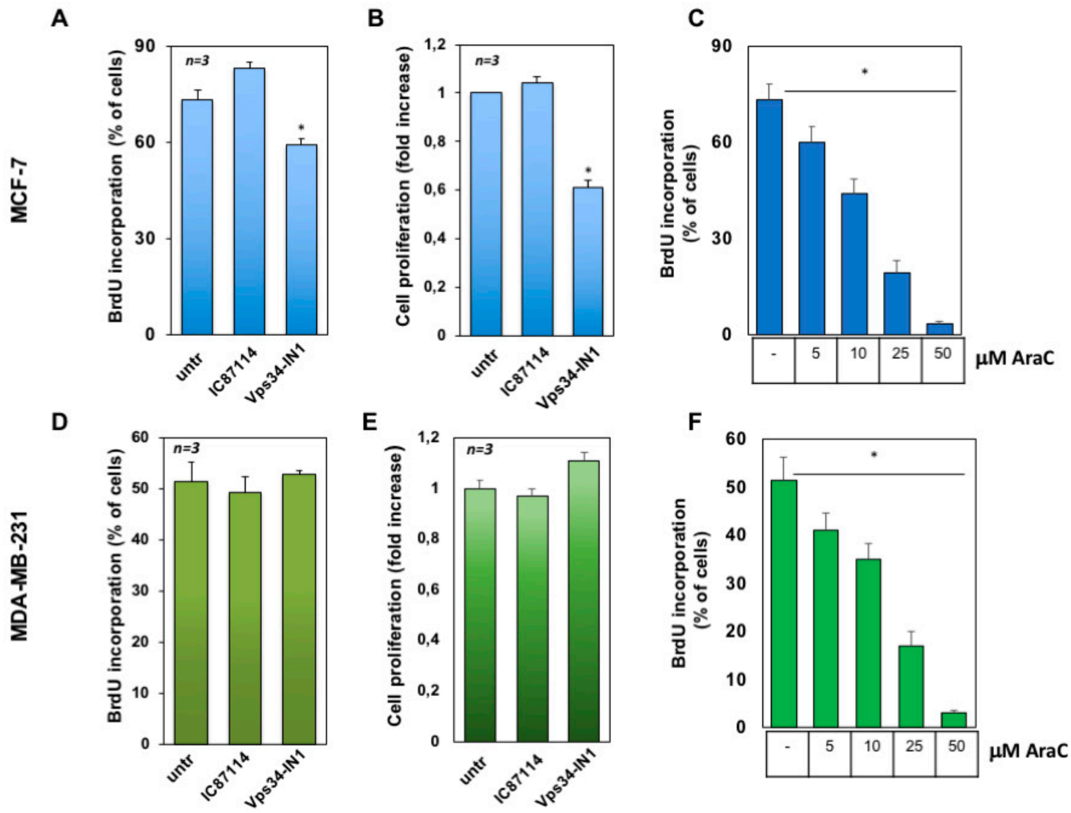

Figure S1: Analysis of BrdU incorporation assay and proliferation in MCF-7 and MDA-MB-231 cells.

Cycling MCF-7 (A and C) and MDA-MB-231 (D and F) cells were left untreated (untr) or treated for 14 h with the indicated inhibitors. Ara-C is the proliferation inhibitor cytosine arabinoside, as indicated in materials and methods section. Cultured cells were *in vitro* pulse-labeled with 100 µM BrdU and its incorporation into DNA was analyzed by immunofluorescence (IF) and expressed as the percentage of total cells. In B and E, cycling MCF-7 (B) and MDA-MB-231 (E) cells were left untreated or treated for 24 h with the indicated inhibitors. Cell proliferation was assayed using the WST-1 reagent. Graphs represent the ratios of proliferation expressed as the fold increase over the basal absorbance. In A-F, Means and SEMs are shown. *n* represents the number of experiments. $^*p <$ 0.05 for the indicated experimental points *vs*. the corresponding untreated control.
